# Branched-Chain Amino Acids Target miR-203a/fosb Axis to Promote Skeletal Muscle Growth in Common Carp (*Cyprinus carpio*)

**DOI:** 10.1155/anu/9406490

**Published:** 2025-02-26

**Authors:** Xianglin Cao, Han Cui, Xinyu Ji, Yaoyajie Lu, Qiuxia Kang, Ronghua Lu, Yuru Zhang, XinXin Xu, Jianjun Chen

**Affiliations:** ^1^College of Fisheries, Henan Normal University, Xinxiang 453007, China; ^2^College of Life Science, Henan Normal University, Xinxiang 453007, China

**Keywords:** branched-chain amino acids, *fosb*, microRNA-203a, skeletal muscle growth and development, starvation stress

## Abstract

Starvation is an environmental stress that cannot be ignored during the growth of aquatic animals. Amino acid composition and balance can influence the nutritional effects, regulating the anabolic metabolism and energy signaling in the organism. Among these, branched-chain amino acids (BCAAs), which are essential amino acids in fish, play vital roles in energy regulation and growth metabolism. In order to observe the recovery effect of BCAAs in sustained duration starvation of common carp, a 4-week starvation period was initiated, followed by a 4-week feeding period with different concentrations of compound BCAAs (leucine:isoleucine:valine = 2:1:1). The detection of skeletal muscle protein deposition, muscle proliferation and differentiation, and indicators related to muscle atrophy at the conclusion of the culture period was conducted. Prior research has demonstrated that microRNA-203a (miR-203a) plays a role in the adaptive regulation of organisms under conditions of energy stress. To further investigate the target-related relationship among BCAAs, miR-203a, and FosB proto-oncogene, AP-1 transcription factor subunit (fosb), an in vitro transfection procedure was conducted in conjunction with topical injections of *fosb* small interfering RNA (siRNA), a miR-203a antagomir, an EEF2K (eukaryotic elongation factor-2 kinase) inhibitor, and an 5-aminoimidazole-4-carboxamide1-*β*-D-ribofuranoside (AICAR) activator. The findings indicated that BCAAs can effectively mitigate muscle damage resulting from starvation, with the 18 g/kg BCAAs diet group demonstrating the most pronounced recovery effect. At the molecular level, BCAAs can regulate the AMPK (adenosine 5‘-monophosphate [AMP]-activated protein kinase) pathway through the miR-203a-mediated targeting of *fosb*, thereby facilitating muscle protein deposition and muscle cell regeneration, ultimately mitigating muscle atrophy. In conclusion, supplementing the diet with BCAAs enhances skeletal muscle protein remodeling by regulating miR-203a, which targets *fosb*. This process promotes the proliferation and differentiation of myoblast, thereby improving the quality of muscle.

**Summary**

• The addition of branched-chain amino acids (BCAAs) to feed supports skeletal muscle protein remodeling.
• FosB proto-oncogene, AP-1 transcription factor subunit (*fosb*) regulates muscle protein synthesis through the AMPK (adenosine 5‘-monophosphate [AMP]-activated protein kinase) pathway.
• microRNA-203a (miR-203a)–*fosb* can regulate myogenesis and protein turnover to improve the muscle mass.

## 1. Background

In response to natural activities such as reproduction, climate change, seasonal alternation, and other environmental stresses, fish utilize a series of physiological, biochemical, and behavioral strategies to regulate organism energetic homeostasis and adaptively regulate their response to starvation stress [1–3]. In order to prioritize the use of energy reserves during starvation, different fish have developed different strategies. Some fish species, such as *Carassius auratus gibelio* [4], *Ictalurus punctatus* [5], and *Danio rerio*, and their larvae [6] are capable of regulating anabolic and catabolic metabolism through the utilization of proteins and lipids as substrates in response to starvation stress at varying time points, including 2–6 weeks or 80 days during overwintering, reproduction, and other circumstances. Nevertheless, subsequent studies on muscle energy metabolism in fasting-treated common carp [7] and Nile tilapia [8], among other species, revealed they self-metabolism and nutrient sensing mobilizing branched-chain amino acids (BCAAs) in skeletal muscle. These findings indicate that prolonged starvation results in muscle atrophy and altered metabolic pathways, which in turn affects energy utilization in fish. The study of starvation can facilitate an understanding of the changes in nutritional requirements of fish during different growth stages and environmental conditions, which is crucial for the development of dynamic feeding strategies.

BCAAs, that is, leucine, isoleucine, and valine, can serve as a source of energy and biosynthesis and also influence intracellular signaling pathways involved in the regulation of nutrient signals which play a unique physiological and biochemical regulatory role in the aquatic animal organism [9]. Unlike other amino acids, the primary site of metabolism for BCAAs is skeletal muscle [10]. Furthermore, appropriate ratios of complex BCAAs have been found to be effective at regulating the proliferation and differentiation of myoblasts [11], the ratio of Leu to Ile to Val should be 0:1 :0.25:0.25, rather than the conventional 1:1:1. Following ingestion or release As feed additives, BCAAs are available in a variety of ratios, including Leu:Ile :Val = 2:1.5 : 1, 2:1:1, 2.2:1.0:1.6, and others. These ratios are suitable for the addition of daily feeds for livestock and poultry, which can enhance the protein value of feeds and facilitate the growth and development of animals [12, 13]. The ratio of Leu:Ile:Val = 1.7:0.8:0.7, which highlights the importance of leucine in the pursuit of muscle growth and the maintenance of a healthy body [14]. Other studies have demonstrated that BCAA combinations (45.8% leucine, 22.9% isoleucine, 27.6% valine) have the capacity to mitigate muscle atrophy and reduce the levels of ubiquitin ligase proteins [15]. Furthermore, this demonstrates the capacity of BCAA supplementation to regulate skeletal muscle protein turnover and myogenesis within the body. BCAAs are transported into the portal vein via small intestinal epithelial cell in organism. They subsequently evade the initial step of amino acid metabolism in the liver, entering the somatic circulation where they are taken up by skeletal muscle and participate in protein synthesis or catabolism in mammals [16, 17]. However, there is a need to further determine the nutritional requirements and utilization of BCAAs by common carp.

During periods of nutrient scarcity, the AMPK (adenosine 5′-monophosphate [AMP]-activated protein kinase) signaling pathway assumes a significant regulatory function within the organism. On the one hand, protein synthesis and cell proliferation were inhibited through lowering the expression of an eukaryotic translation elongation factor 2 (eef2), mTORC1 (mammalian target of rapamycin complex 1). On the other hand, the ubiquitin proteasome pathway (UPP) and autophagy pathway were regulated to change protein catabolism [18, 19]. Prior research has demonstrated that BCAAs can also inhibit the AMPK pathway, thereby promoting metabolic homeostasis in the body [20]. Accordingly, this study investigated whether exogenous BCAAs could reinstate protein turnover in starved common carp, facilitate muscle synthesis, and maintain skeletal muscle energy balance, and basic function under conditions of nutrient deprivation. FosB proto-oncogene, AP-1 transcription factor subunit (FosB) is involved in the regulation of mammalian myoblast proliferation, differentiation, and transformation, as well as a key factor in the repair process of muscle damage [21, 22]. Despite the absence of direct evidence linking the FosB to BCAA metabolism, our previous studies transcriptome and small RNA sequencing studies indicate that BCAAs (Leu:Ile:Val = 2:1:1) can participate in the regulation of skeletal muscle protein remodeling and skeletal myogenesis by regulating DGMs, such as microRNA-203a (miR-203a) and miR-192, and by targeting genes *fosb*, *mef2c* (myocyte enhancer factor 2C), etc., involved in proteolysis metabolism and myocyte proliferation and differentiation. Of particular, interests were the substantial alterations in the gene expression levels of the target gene, FosB, as predicted by miR-203a. [23]. It was shown that MyoG (myogenin) enhances sponge adsorption of miR-203a by circGPD2 to deregulate its inhibition of *c-Jun* (Jun proto-oncogene, AP-1 transcription factor subunit) and *mef2c*, ultimately promoting myoblast proliferation and differentiation. In contrast, miR-192 regulates the proliferation and myogenic differentiation of sheep satellite cells (SCs) by targeting retinoblastoma 1 (RB1), thereby affecting muscle development. These findings also indicate that microRNAs (miRNAs) play a crucial role in the growth, development, and nutrient regulation of skeletal muscle in common carp. Significant changes in gene expression levels of *fosb*, a target gene predicted by miR-203a, were found after the addition of exogenous BCAAs to starved common carp [23]. However, the mechanism by which BCAAs affect common carp endogenous miRNAs to regulate skeletal muscle development in the organism is rarely reported. Therefore, we will use miR-203a and *fosb* as an entry point to investigate whether this is a key regulator of carp skeletal muscle growth and development.

The aim of this study was to verify the effect of BCAAs on the growth process of starved common carp and to explore the potential functions of BCAAs in common carp skeletal muscle. This research establishes a foundation for optimizing BCAAs application in aquaculture, while also delineating the regulatory mechanisms governing skeletal muscle growth and development in freshwater fish species.

## 2. Methods

### 2.1. Ethical Statement

The animal-related procedures within this study adhered to the ethical standards as delineated in the Guidelines for the Care and Use of Laboratory Animals, in accordance with the Regulations for the Administration of Laboratory Animal Affairs. Furthermore, all experimental protocols were duly approved by the Animal Ethics Committee of Henan Normal University (Approval No: HNSD-2023BS-0428).

### 2.2. Experimental Animals

Healthy common carp (58.4 ± 2.0 g; 14.90 ± 0.50 cm) from Xingda Aquaculture Farm, Jinshui District, Zhengzhou City, Henan Province, China, were used in the experiments. A total of 360 fish were randomly assigned to four distinct groups, with each group comprising three replicates. The common carp were transferred to an 80 × 120-cm-diameter temperature-controlled recirculating water culture system, fed under stable feeding conditions for 2 weeks of acclimatization, and then used for the formal experiments. After 4 weeks of starvation, the common carp were fed diet (3% body weight) supplemented with 0, 16, 18, or 20 g/kg complex BCAAs (leucine:isoleucine:valine = 2:1:1; Solepol, China; 10.7% lipids and 34.9% proteins on a dry matter basis) for a period of 30 days. The compound BCAAs were added into the premix feed powder, and the pellet feed with a diameter of 2.5 mm was made after mixing, dried, formed, packed, and refrigerated at −20°C (Table 1). The crude protein, crude fat, and moisture data in the experiment were conducted following the protocols outlined by the Association of Official Analytical Chemists [24]. During the acclimatization and formal experiments, common carp were fed three times per day (8:00, 12:00, and 18:00) for a period of 30 days. The water temperature was maintained at 23 ± 2°C, the pH was 7.2 ± 0.12, the dissolved oxygen concentration was 5.9 ± 0.3 mg/L, the ammonia nitrogen concentration was <0.01 mg/L, the water hardness (230 mg of CaCO_3_/L) was 42.5 ± 1.2 mg/L Ca^2+^, and the light/dark photoperiod was 12 h/12 h. At the end of the experimental period, the fish were anesthetized with 100 mg/L tricaine methane sulfonate (MS-222, Buxi Chemical, China) and weighed, and skeletal muscle tissue samples were collected from the posterior edge of the gill cover to the anterior edge of the caudal fin were taken for subsequent experiments. A portion of the tissues was snap-frozen in liquid nitrogen, and small pieces of the tissues were placed into 1.5 mL EP tubes, added to RNAiso Plus (Takara, Japan), and then placed in a refrigerator at −80°C for later preservation.

The cells used in the experiment were 293T cells (laboratory preserved). Cell cryopreserving was subjected to cell resuscitation and passaging culture in cell culture flasks in a sterile environment. The procedure was performed using Dulbecco's modified eagle medium enriched with 10% fetal bovine serum in a constant temperature incubator at 37°C with 5% CO_2_. Before the transfection process, 293T cells were seeded into six-well plates and allowed to grow to a suitable cell density for the experiments that were to follow.

### 2.3. Tissue Morphology Analysis

Upon the conclusion of the feeding phase, the skeletal muscle was cut into tissue slices with a size of 1 cm × 1 cm × 0.2 cm and fixed incubation in Bouin's solution (Servicebio, China), rinsed in running water. The slices were then removed, rinsed twice with 1 × PBS, dehydrated for 1 h in 70%, 80%, 95%, and 100% ethanol gradients, and soaked in xylenes I and II for 30 min; the removed tissue blocks were immersed in paraffin wax solution at ~50°C and then in another wax solution for 1.5 h and embedded in an embedding machine. The carrier drag and the tissue block were stuck tightly and then fixed in the slot of the frozen sectioning machine (RM2235, Rongxing Guangheng, China). The angle and thickness were adjusted for slicing and spreading, and the slices were then baked for 30 min at 60–65°C and stored at room temperature.

The Masson staining method initially involved chroming the slices, followed by immersion in a saturated picric acid (Servicebio, China) alcohol bath to eliminate formalin pigment and subsequent rinsing with distilled water. After thorough drying, the slides were stained with Weigert's hematoxylin (Biyuntian, China) dye solution for 10 min. Following complete washing with water, the next step involved staining with Masson's ponceau acid red dye (Servicebio, China) at room temperature for 10 min once the cell nuclei appeared blue under microscopy. Poststaining, a 0.2% glacial acetic acid aqueous solution (Servicebio, China), was employed thrice for 1 min each time to remove any residual red floating coloration. Subsequently, a 1% phosphomolybdic acid aqueous solution (Servicebio, China) was added for differentiation and treatment lasting 3 min; excess liquid was then blotted using filter paper before immersing the slide in aniline blue dyeing solution (Servicebio, China) for 10 min. Finally, cleaning of the slide with a 0.2% acetic acid aqueous solution ensured clear and vibrant coloring. The dehydration step was repeated, and the sections were sealed with neutral resin and analyzed microscopically using ImageJ software.

### 2.4. Immunohistochemical Staining

The paraffin-embedded sections were deparaffinized, hydrated, and then antigenically repaired using EDTA. Endogenous peroxidase was added to the sections for 10 min to block the reaction, then the sections were washed on a shaker for 5 min and repeated three times. Subsequently, the sectioned tissues were incubated with goat serum blocking solution, incubated with primary anti-eEF2 (eukaryotic translation elongation factor 2) (Novus Biologicals, China), anti-FosB rabbit pAb (Servicebio, China), and goat antirabbit secondary antibody (Servicebio, China), and then incubated with horseradish enzyme-labeled streptavidin (Thermo Fisher Scientific, China) to cover the sectioned tissue. The tissues were then subjected to color development using DAB chromogenic solution (Servicebio, China), hematoxylin restaining, differentiation, dehydration, and sealed with neutral resin. After microscopic observation, the results were analyzed and quantified by determining the average optical density (AOD) using ImageJ software.

### 2.5. Small Interfering RNA (siRNA) Transfection

Preliminary experiments involving in vivo siRNA transfection were first performed. The control group received a treatment of 0.65% saline solution, and si-NC, si-*fosb-3*4, si-*fosb*-680, and si-*fosb*-1124 were mixed with Entranster-in vivo (Engreen Biosystem, China). The siRNA primer sequences were as follows: si-NC-F/R, 5′-UUCUCCGAACGUGUCACGUTT-3′, and 5′-ACGUGACACGUUCGGAGAATT-3′; si-*fosb*-34-F/R, 5′-GACGAAGACUCCAAGUAAATT-3′, and 5′- UUUACUUGGAGUCUUCGUCTT-3′; si-*fosb*-680-F/R, 5′-GACGAAGACUCCAAGUAAATT-3′, and 5′-UUUACUUGGAGUCUCUUCGUCTT-3′; and si-fosb-1124-F/R, 5′-CCCUCAUACACAUCUUCAUTT -3′, and 5′-AUGAAGAUGUGUAUGAGGGTT-3′ (GenePharma, China). The skeletal muscle samples of common carp in the different treatment groups were injected with 50 μL of 11 μg/tail of *fosb* siRNA or 0.65% saline after 24 h or 48 h, respectively. The skeletal muscle tissues were collected in a sterile environment for snap freezing and stored in a −80°C freezer, and 1cm × 1cm × 1 cm tissue blocks were collected, fixed with 4% paraformaldehyde, and then stored at 4°C in a refrigerator.

### 2.6. Drug Treatment and miR-203a Antagomir Treatment

In the control cohort, the skeletal muscle was administered with 0.65% saline, and 500 µL of 50 mg/kg/day 5-aminoimidazole-4-carboxamide1-*β*-D-ribofuranoside (AICAR) (MedChemExpress, China) was locally injected into the skeletal muscle tissue of the treated groups of common carp. At the same time, after 1 week of treatment without or with 18 g/kg BCAAs, 50 µLsi-NC or 11 μg/tail *fosb* siRNA (si-*fosb*-680) were cotreated, respectively, and skeletal muscle tissue samples were collected after 24 h.

In addition, an intramuscular injection of 500 µL of 1 mg/kg/day NH125 (Macklin, China) was administered. The following groups were established: 0.65% saline group, NH125 group, NH125 recovery group (fed a diet with 18 g/kg BCAAs), and the NH125+*fosb* siRNA group (fed a diet with 18 g/kg BCAAs). Similarly, 1 week after NH125 injection, the common carp were subjected to *fosb* siRNA treatment, and samples were collected at the end of the experiment.

In vivo treatment with the miRNA antagomir (Sangon Biotech, China) was conducted by topical administration (0.5 nmol/day intramuscular injection) of 50 µL, and this treatment was conducted for both the 0.65% saline control group and the antagomir NC control group. Four days after the injection treatments, common carp in the control and miRNA antagomir groups were injected with 11 μg/tail si-NC and *fosb* siRNA, respectively, and samples were collected 24 h later. The antagomir-203a sequence was 5′-(mC)*⁣*^*∗*^ (mA)*⁣*^*∗*^ (mA) (mG) (mU) (mG) (mG) (mU) (mC) (mC) (mU) (mA) (mA) (mA) (mC) (mA) (mU) (mU) (mU)*⁣*^*∗*^ (mC)*⁣*^*∗*^ (mA)*⁣*^*∗*^ (mC)*⁣*^*∗*^ (Chole)-3′, and the antagomir NC sequence was 5′-(mC)*⁣*^*∗*^ (mA)*⁣*^*∗*^ (mG) (mU) (mA) (mC) (mU) (mU) (mU) (mU) (mG) (mU) (mG) (mU) (mA) (mG) (mU) (mA)*⁣*^*∗*^ (mC)*⁣*^*∗*^ (mA)*⁣*^*∗*^ (mA)*⁣*^*∗*^ (Chole)-3′.

### 2.7. Bioinformatics and Dual-Luciferase Reporter Assay System

For miR-203a and *fosb* 3′-UTR target binding site prediction, the miR-203a and *fosb* 3′-UTR target binding sites were analyzed using miRanda software (available at http://www.microrna.org), RNAhybrid 2.2 (available from RNAhybrid), and TargetScanFish (available at https://www.targetscan.org/fish_62/).

According to the binding site of miR-203a target gene *fosb* 3′UTR in TargetScan online program, the 250 bp sequence of the binding region was synthesized and amplified by specific primers. The cloning vector PMD-19T was used for sequence cloning, and the restriction sites of SacI and SalI (CWBIO, China) were selected for subsequent gel recovery and double digestion. After sequencing alignment, *fosb* 3′UTR-WT was obtained and synthesized (BGI, China). Then, they were cloned into pmirGLO Dual-Luciferase miRNA Target Expression Vector (Promega, USA). A segment of the 3′UTR of the target gene *fosb* was successfully cloned into the 3′ terminus of the dual-luciferase expression vector, *Luciferase2*. The resultant construct was designated as PmirGLO-*fosb* 3′UTR-WT. The mutant sequence was named PmirGLO-*fosb* 3′UTR-Mut after connecting to the vector. The process of double digestion, ligation, transformation, and plasmid extraction followed the optimization steps provided in the kit instructions (CWBIO, China).

Subsequently, 20 pmol miR-203a mimics and NC were diluted with 10 μmol Opti-MEM medium, and 5 ng *fosb*-WT, *fosb*-Mut plasmid and 10 ng pmirGLO vector reference plasmid was diluted with 10 μmol Opti-MEM medium, respectively. Finally, the diluted Lipofectamine 3000 (Thermo Fisher Scientific, USA) was added dropwise to each mixed solution placed in groups, gently mixed, and allowed to stand at room temperature for 5–10 min to form a transfection complex. When 50 μL transfection complex was added to the culture plate, it was gently shaken to show uniform distribution in human embryonic kidney cells (cells stored in the laboratory). Three control groups were set up in each group, and then the cells were placed back in the cell incubator and cultured at 37°C and 5% CO_2_. After 48 h of transfection, the dual-luciferase detection kit was used to detect the intracellular luciferase activity using a multifunctional microplate reader according to the instructions to evaluate the effect of the interaction between miR-203a and *fosb* 3′UTR on luciferase gene expression.

### 2.8. RNA Isolation and Quantitative RT-PCR

Skeletal muscle tissue samples were ground, and total RNA was extracted with TRIzol reagent (Invitrogen, USA). The quality and concentration of the extracted RNA were detected by measuring the absorbance value at A260/A280 using a NanoDrop Spectrophotometer, and the integrity of the total RNA samples was assessed by agarose gel electrophoresis. We designed the primer sequences using GenBank (http://www.ncbi.nlm.nih.gov/), and the primers were synthesized by Beijing Genomics Institute (BGI, Shenzhen, China). In the experiments, *rpl8* and *U6* were used as the internal reference genes for messenger RNA (mRNA) and miR-203a, respectively. The miRcute Plus miRNA first-strand cDNA Kit (TianGen, China) and the PrimeScript Reverse Transcriptase Kit (Takara, Japan) were used for reverse transcription of miRNA first-strand cDNA and cDNA synthesis, respectively. The first-strand of miRNA cDNA was reverse transcribed, and cDNA was synthesized using AceQ qPCR. For miRNA first-strand cDNA, the following temperature program was used: 42°C for 60 min and 95°C for 3 min. The products were stored in a refrigerator at −20°C. mRNA reverse transcription was added and mixed using 20 μL of reverse transcription system. The protocol involved a 15 min incubation period at a temperature of 37°C, followed by denaturation at 85°C for 5 s, and then holding at 4°C for an extended period. Real-time quantitative reverse transcription polymerase chain reaction (qRT-PCR) was performed using a LightCycler 480 II instrument (Roche, Switzerland) with 50 μL of the following mixture (25 μL of 2 × UltraSYBR mixture, 1 μL each of the upstream and downstream primers, 2 μL of the cDNA template, and 21 μL of ddH_2_O). The following steps were used for transcription: initial denaturation at 95°C for 10 min, 40 cycles of 95°C for 15 s and 60°C for 60 s and melt curve at 95°C for 15 s, 60°C for 60 s, 95°C for 15 s, and 60°C for 15 s. For miRNA fluorescence quantification, a 10-µL reaction system was used (5 µL of 2 × miRcute Plus miRNA PreMix, 0.2 µL of each primer, 1 µL of cDNA, and 3.6 µL of ddH_2_O). The following reaction program was established: template denaturation at 95°C for 15 min followed by annealing at 94°C for 20 s and 60°C for 34 s via 40 cycles of extension. All the results were analyzed using the 2^−*ΔΔ*CT^ method (Livak method). The relevant primer sequences are listed in Table S1.

### 2.9. Statistical Analysis

The statistical data were analyzed using SPSS 22.0 software and GraphPad Prism 5.0 software (GraphPad Software, Inc., USA). Student's *t*-test was used to compare two treatment groups, one-way analysis of variance (ANOVA) was used for the comparison of at least three treatment groups, and Tukey's test was then used for multiple comparisons and for generating the graphs. The values are expressed as the means ± standard deviations (means ± SDs). A *p* value < 0.05 indicated that the difference was statistically significant.

## 3. Results

### 3.1. BCAAs Regulate Skeletal Myogenesis in a Dose-Dependent Manner

**Figure 1:**
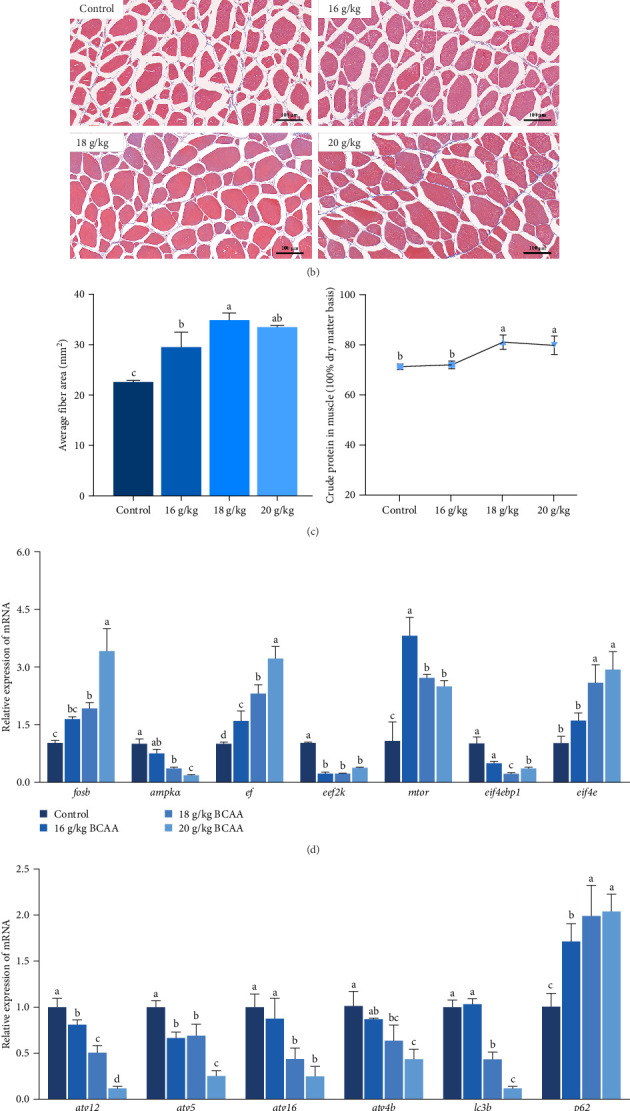
Effects of the dietary BCAAs levels on skeletal muscle production in starved common carp. (A) Mean body weight change (*n* = 6) of common carp over an 8-week period and percentage of crude protein, crude fat, and moisture content of the diet. (B) Masson staining of skeletal muscle of common carp. (C) Quantification of the area of skeletal muscle myofibers and the percentage of crude protein. The relative expression of genes related to protein synthesis (D), autophagy (E), and myogenesis regulator and cell proliferation (F) were detected by qRT–PCR. Values for statistical analyses are presented as mean ± SD (*n* = 6), while different letters are used to represent significant differences between groups. BCAAs, branched-chain amino acids; qRT–PCR, quantitative reverse transcription polymerase chain reaction.

In this study, we first investigated whether BCAAs could participate in the nutritional regulation of starved common carp to alleviate metabolic stress or muscle damage. In these experiments, we used diets with no significant differences in crude protein, crude fat, or moisture content for satiation (Figure 1A) and found that poststarvation refeeding induced histomorphometric changes in the skeletal muscle tissue of starved common carp, such as myocyte atrophy and an increase in the gap between muscle bundles (Figure 1B). Notably, the group administered 18 g/kg BCAAs exhibited the most significant elevation in average body weight 2 weeks postfeeding resumption. By the eighth week, this group's average body weight notably surpassed that of the other experimental groups (Figure 1A). Moreover, 18 g/kg BCAAs group had significantly higher muscle crude protein and muscle fiber area than the control group (Figure 1C). Therefore, 18 g/kg BCAAs (10.7% lipids and 34.9% proteins) significantly increased the protein utilization in skeletal muscle and promoted the growth of common carp.

Following that, we conducted a detailed analysis to measure protein synthesis within the skeletal muscle and the expression levels of genes associated with autophagy. These factors collaboratively influence the overall protein balance and the rate of muscle protein turnover in the skeletal muscle tissue. The results revealed that the BCAAs-treated group exhibited significantly upregulated *mtor*, eukaryotic translation initiation factor 4E (*eif4e*), and *ef* expression (*p* < 0.05) (Figure 1D) and significantly downregulated *ampk*, *eef2k* (eukaryotic elongation factor-2 kinase), and *eif4ebp1* (eukaryotic translation initiation factor 4E binding protein 1) expression (*p* < 0.05) (Figure 1D). Furthermore, there was a notable reduction in the expression of autophagy-related genes, including *atg12* (autophagy related 12), *atg5* (autophagy related 5), *atg16* (autophagy related 16), *atg4b* (autophagy related 4b), and *lc3b* (microtubule associated protein 1 light chain 3 beta [MAP1LC3B]), in comparison to the control group (*p* < 0.05) (Figure 1E). Concurrently, a significant increase was observed in the expression of the nucleoporin 62 (*p62)* gene (*p* < 0.05). These results suggest that dietary BCAAs can coordinate protein synthesis and starvation-induced autophagy.

In addition, we examined the skeletal muscle development-related genes *myod1* (myogenic differentiation 1), *myog*, *myhc* (myosin heavy chain 6), *cyclind1*, and *pcna* (proliferating cell nuclear antigen) (Figure 1F) and found that the expression levels of *myod1* and *myhc* were significantly increased in the 16 and 18 g/kg BCAAs groups (*p* < 0.05). The expression level of *cyclind1* was significantly upregulated in the experimental groups (*p* < 0.05), and that of *pcna* was significantly upregulated only in the 18 g/kg BCAAs group (*p* < 0.05). The abovedescribed results indicated that BCAAs could increase the expression of genes related to myoblast proliferation and differentiation to a certain extent.

### 3.2. BCAAs Regulate *fosb* Expression and Promote Skeletal Muscle Growth

Fos family proteins are regulators of cell proliferation, differentiation, and transformation and can also participate in the repair of muscle damage [21, 25]. Utilizing transcriptomic and small RNA sequencing data from our prior research, it was observed that the introduction of BCAAs notably alters the transcriptional activity of the skeletal muscle *fosb* gene. Leveraging this observation, we have adopted *fosb* as a pivotal entry point to investigate its function as a regulatory element downstream of BCAAs in the process of myogenesis.

**Figure 2:**
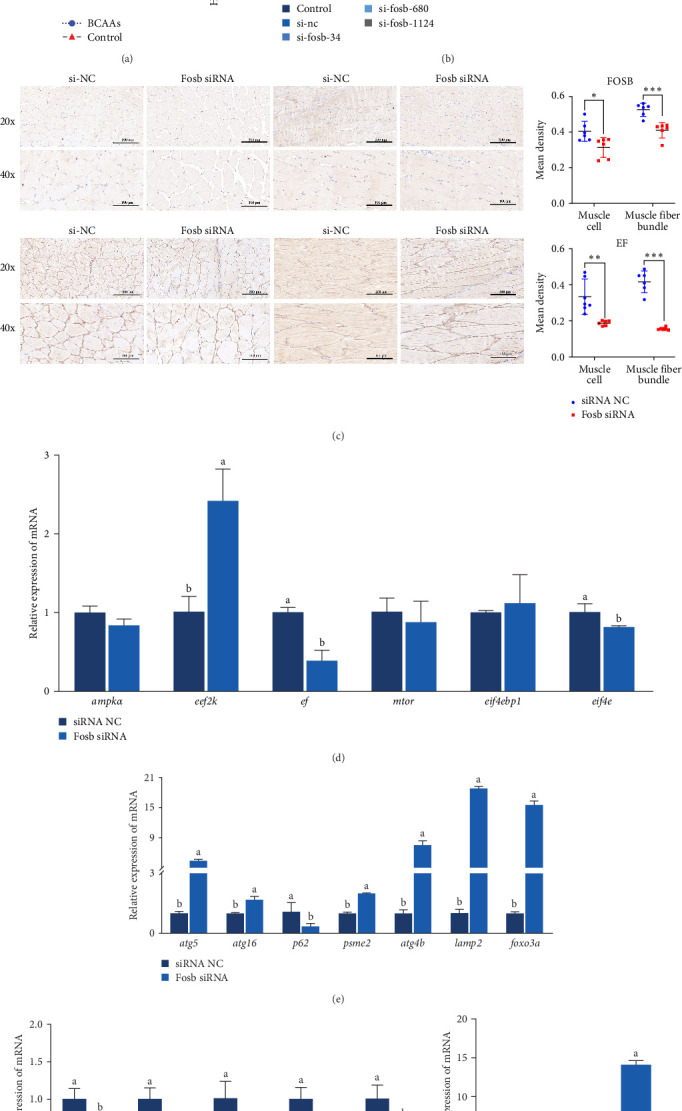
*fosb* is involved in the amelioration of starvation-induced skeletal muscle damage via BCAAs. (A) Relative expression of *fosb* in different tissues. (B) Interference effects of *fosb* siRNA at different time points. (C) Immunohistochemistry of skeletal muscle for FOSB and EF, and quantification of AOD values and protein levels by observing transverse and longitudinal sections of muscle fibers at 20x and 40x , respectively. The relative expression of genes related to protein synthesis (D), autophagy (E), muscle regulatory factors and cell proliferation (F), and muscle atrophy (G) were examined. Tukey's test post hoc multiple comparisons were performed on a series of data by mean ± SD (*n* = 6), and significant difference letter marking method was used to differentiate differences in expression (*p* < 0.05); *⁣*^*∗*^*p* < 0.05, *⁣*^*∗∗*^*p* < 0.01, *⁣*^*∗∗∗*^*p* < 0.001 vs. si-NC group. BCAAs, branched-chain amino acids; fosb, FosB proto-oncogene, AP-1 transcription factor subunit.

The expression of *fosb* in skeletal muscle, brain, and liver tissues of the experimental groups was significantly higher than that in the control group (Figure 2A). Subsequent interference of *fosb* in skeletal muscle tissues revealed that si-*fosb*-680 exerted the best interference effect (*p* < 0.05), and this siRNA was thus used in subsequent experiments (Figure 2B). *fosb* siRNA interference was used to quantify the AOD of the transverse and longitudinal morphology of the observed skeletal muscle tissues and the level of FOSB protein, and the results showed that *fosb* interference significantly reduced the FOSB protein level in the myofibers (*p* < 0.05), causing myocyte atrophy and an increased myofibrillar gap (Figure 2C). Subsequently, genes related to the AMPK-EEF2K pathway and the mTOR-EIF4E pathway were examined, and no significant differences in the expression of *ampk*, *eif4ebp1*, or *mtor* were found after siRNA interference (*p* > 0.05); however, these genes could significantly reduce the expression of *ef* and *eif4e* and significantly increase the expression of *eef2k* (Figure 2D). Additionally, we found that *fosb* interference could significantly reduce the FOSB protein level in muscle fibers (*p* < 0.05) (Figure 2C), causing muscle cell atrophy and increasing the gap between muscle bundles. We also found that the level of EF protein in muscle fibers was significantly reduced (*p* < 0.05) (Figure 2C). Therefore, we propose that *fosb* may act as a downstream regulator of AMPK and regulate muscle protein synthesis through the AMPK-EEF2K pathway rather than the mTOR pathway.

Further experimental data revealed that the suppression of fosb by siRNA notably elevated the expression of autophagy-associated genes such as *atg5*, *atg16*, *atg4b*, *psme2* (proteasome activator subunit 2), *lamp2* (lysosomal associated membrane protein 2), and *foxo3a* (forkhead box O3) (*p* < 0.05) (Figure 2E). Conversely, it led to a significant decrease in the expression of *p62* and in genes associated with muscle differentiation and proliferation, including *myod1*, *myog*, *myhc*, *cyclind1*, and *pcna* (*p* < 0.05) (Figure 2F). Moreover, the *fosb* siRNA group exhibited significantly increased expression of the muscle atrophy-related genes *atrogin1* and *murf1* (tripartite motif containing 101) (*p* < 0.05) (Figure 2G). The abovementioned results demonstrated that dietary BCAAs could regulate the involvement of *fosb* in skeletal muscle protein synthesis in starved common carp and that FOSB, in addition to its role in regulating myogenesis, could be involved in the regulation of autophagic flux and the maintenance of skeletal muscle protein homeostasis.

### 3.3. *fosb* Regulates Muscle Protein Synthesis Through the AMPK-EEF2K Pathway

**Figure 3:**
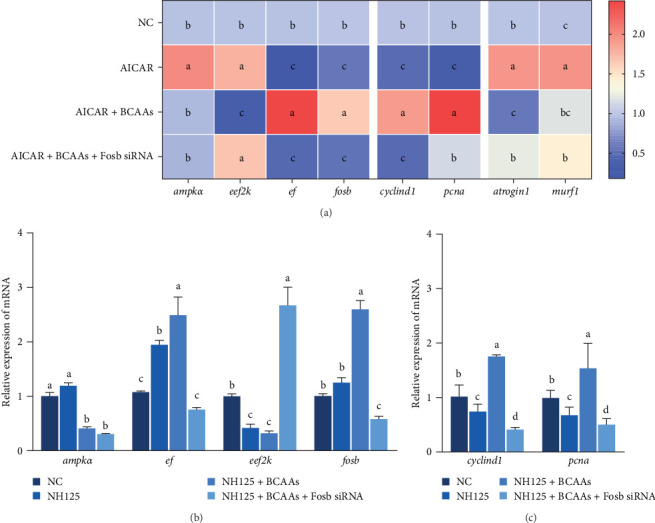
*Fosb* regulates muscle protein synthesis through the AMPK-EEF2K pathway. The relative expression of genes related to AMPK-EEF2K pathway, myocyte proliferation, and atrophy (A) was examined in the AMPK activator AICAR-treated group. The relative expression of genes related to AMPK-EEF2K pathway (B), myocyte proliferation (C) was detected after treatment of common carp with eef2k inhibitor NH125. Data are mean ± SD (*n* = 6), and significant differences were differentiated using the significant difference letter labeling method to distinguish whether the differences between groups were significant (*p* < 0.05). fosb, FosB proto-oncogene, AP-1 transcription factor subunit.

AMPK, when activated as a highly conserved energy sensor, is capable of limiting energy expenditure, that is, through proliferation or protein synthesis, and the body's catabolic processes, that is, glycolysis or fatty acid oxidation [26–28]. The subsequent studies were designed to delve deeper into the role of *fosb* within the AMPK-EEF2K signaling axis, which is implicated in the control of protein synthesis. It is well-documented that the activity of EEF2K is sensitively modulated by nutrient availability, with AMPK being a key positive regulator in this context. We therefore treated common cart with the AMPK activator AICAR. The AICAR-treated group exhibited significantly increased expression of *ampkα* (*p* < 0.05), but no significant change was detected in the other groups (*p* > 0.05) (Figure 3A). Both AICAR treatment alone and cotreatment with BCAAs and *fosb* siRNA significantly suppressed the expression of *fosb* and *ef*, and BCAAs recovery reversed the suppressive effect of *ampk* on the expression levels of *fosb* and *ef*, whereas the expression of *eef2k* showed a completely opposite trend to that of *fosb* and *ef* (*p* < 0.05) (Figure 3A).

We further treated common carp with the EEF2K inhibitor NH125 and found that this treatment resulted in a marked decrease in the transcription of the *eef2k* gene, while it upregulated the expression of the *ef* gene (*p* < 0.05). Cotreatment with BCAAs further an enhancement in the transcriptional activity of *fosb* and *ef* genes. This methodological strategy also resulted in the downregulation of *ampk* and *eef2k* genes (*p* < 0.05). Treatment with the EEF2K inhibitor or cotreatment with BCAAs and *fosb* siRNA significantly downregulated the *ef* and *fosb* expression levels, whereas the opposite trend was found for *eef2k* expression (*p* < 0.05) (Figure 3B).

We also found that supplementation with BCAAs restored the negative regulation of cell proliferation-related genes by NH125 and AICAR agonists, and this effect was reversed after *fosb* interference (*p* < 0.05) (Figure 3A,C). Interestingly, the expression of muscular atrophy-related genes was affected by the AICAR agonist and *fosb* siRNA in the same manner. The abovedescribed results suggest that BCAAs can regulate muscle mass through a mechanism involving the downstream regulator *fosb* in the AMPK-EEF2K pathway (*p* < 0.05) (Figure 3A).

### 3.4. miR-203a Targets *fosb* to Regulate Translation Elongation

**Figure 4:**
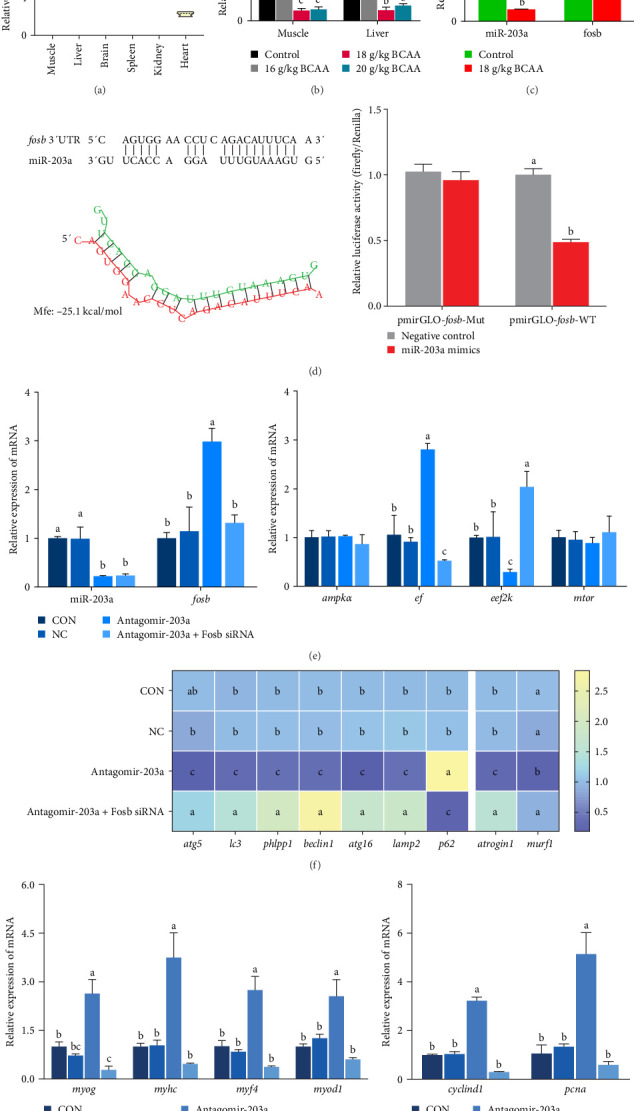
miR-203a targets *fosb*, which is involved in the BCAA-mediated regulation of the skeletal muscle growth process. (A) miR-203a expression in different tissues. (B) Relative expression of miR-203a in hepatopancreas and skeletal muscle after branched-chain amino acid addition. (C) Changes in relative expression trends of miR-203a and *fosb* after branched-chain amino acid addition. (D) The targeting sites of miR-203a and fosb were predicted and verified by dual-luciferase reporter gene assay. (E) To detect the expression of miR-203a, *fosb*, and AMPKA-EEF2K pathway-related genes in skeletal muscle after miR-203a antagomir treatment. As well as the expression of genes related to autophagy and atrophy (F), myogenesis regulator (G), and myocyte proliferation (H) in skeletal muscle after miR-203a antagomir treatment. The form of data used for statistics is mean ± SD (*n* = 6), and different letters were used to represent significant differences between groups. fosb, FosB proto-oncogene, AP-1 transcription factor subunit.

miR-203 is widely recognized as a tumor suppressor, but recent studies have shown its involvement in the regulation of muscle development [29, 30]. In this previous research, we found that the supplementation of BCAAs significantly affects the expression of noncoding RNAs such as miR-203a in common carp skeletal muscle. Here, we explored the interactions between BCAAs and *fosb* or miR-203a. First, we examined the relative expression of miR-203a in different tissues and found that miR-203a expression was higher in muscle tissues than in other tissues and was significantly decreased with increases in the BCAAs concentration (*p* < 0.05) (Figure 4A,B). Subsequently, we found that the expression of *fosb* was higher in the group supplemented with the medium concentration than that in the control group, whereas the expression of the miR-203a gene exhibited the opposite change to that found for the *fosb* gene (*p* < 0.05) (Figure 4C). We subsequently identified the target sites of action of both *fosb* and miR-203a via bioinformatics analysis. The results of dual-luciferase reporter gene assay showed that there was no significant change in the fluorescence intensity of NC and PmirGLO-*fosb* 3′UTR-WT and PmirGLO-*fosb* 3′UTR-Mut coprocess groups, while miRNA mimics and PmirGLO-*fosb* 3′UTR-WT and PmirGLO-*fosb* 3′UTR-Mut cotreatment groups. The fluorescence intensity of PmirGLO-*fosb* 3′UTR-WT group was significantly decreased (*p* < 0.05) (Figure 4D). The above results indicate that miR-203a has a direct targeting binding effect with *fosb*.

To further observe the effect of miR-203a on protein synthesis and catabolism as well as myocyte proliferation and differentiation in common carp skeletal muscle, we injected antagomir-203a into skeletal muscle tissues to inhibit the expression of miR-203a. Detection of the expression of skeletal muscle protein synthesis-related genes after miR-203a antagomir treatment showed that antagomir-203a could significantly increase the expression of the *fosb* and *ef* and significantly inhibit the expression of the *eef2k* (*p* < 0.05) (Figure 4E). In addition, cotreatment with antagomir-203a and *fosb* siRNA significantly decreased the expression levels of the *fosb*, *ef* genes, and significantly increased the expression of *eef2k* (*p* < 0.05), whereas cotreatment with fosb siRNA did not affect the expression of miR-203a compared with that found after treatment alone. The results showed that the *ampk* and *mtor* genes, as upstream regulators, were not significantly affected, regardless of the change in miR-203a with respect to *fosb*.

The potential role of miR-203a in the autophagy of skeletal muscle cells was also investigated. We detected the expression of the autophagy-related genes *atg5*, *atg16*, *lc3*, *phlpp1* (PH domain and leucine rich repeat protein phosphatase 1), *lamp2*, *beclin1*, and *p62* (Figure 4F). The results revealed that antagomir-203a significantly inhibited the expression of all the tested genes except *p62*, and these effects were reversed by *fosb* siRNA cotreatment (*p* < 0.05). In contrast, in the Antagomir-203a group, the gene expression levels of *atrogin1* and *murf1* were significantly inhibited and were significantly upregulated in the Antagomir-203a and Fosb siRNA cotreatment group (*p* < 0.05). These findings suggest that the suppression of miR-203a expression can significantly reduce the autophagy pathway and alleviate muscle atrophy to some extent.

In this study, the expression of genes related to muscle differentiation and proliferation was found to be significantly increased in the skeletal muscle tissues of the antagomir-203a antagomir-treated group (Figure 4G,H). As expected, although miR-203a expression was suppressed at this time point, the expression of these genes, which might be involved in muscle development and promote myoblastogenesis and myofibril transformation and fusion, was also reduced after the *fosb* gene was knocked down (*p* < 0.05). These results suggest that BCAAs regulate miR-203a to target *fosb* and thus improve the muscle mass in common carp.

## 4. Discussion

Understanding the composition of fish is particularly important for the efficient utilization of dietary amino acid patterns. Research has increasingly focused on strategies for formulating feeds with amino acids to achieve improved feed production performance through precise quantitative analysis [31]. BCAAs include three essential BCAAs, namely, leucine, isoleucine, and valine. A review of the literature reveals that the optimal concentrations of dietary BCAAs for Indian major carp (*Cirrhinus mrigala*, Hamilton) are valine, isoleucine, and leucine, with a concentration of 15.2 g/kg, 12.6 g/kg, and 15.4 g/kg, respectively, corresponding to 38.0 g/kg, 31.5 g/kg, and 38.5 g/kg of dietary protein, respectively [12]. The dietary isoleucine requirement of juvenile Indian major carp (*Labeo rohita*, Hamilton) was found to be 15.2–15.9 g/kg dry feed, corresponding to 38.0–39.8 g/kg dietary protein. This is similar to the results obtained in another study, which reported a requirement of 1.50–1.57 g per 100 g dry feed, corresponding to 3.75–3.92 g per 100 g dietary protein [32, 33]. The optimal concentration of leucine in rainbow trout (*Oncorhynchus mykiss*) was determined to be 17.45 g/kg, corresponding to 38.77 g/kg dietary protein. This concentration was found to facilitate optimal growth of rainbow trout juveniles and to regulate the expression of genes related to the TOR and 4E-BP signaling pathways [9]. The results suggest that BCAAs can enhance growth performance and feed efficiency in fish organisms.

Additionally, previous studies on BCAAs have primarily focused on the activation of mTORC1 by single BCAAs via amino acid sensors. Further investigation on the concentration of different ratios of BCAAs as functional additives after matching is necessary. Previous studies on BCAAs have focused on the activation of mTORC1 by a single BCAAs via an amino acid sensor. The activity of mTORC1 is enhanced by Leucine through the GATOR2-GATOR1-KICSTOR-Rags signaling axis, following detection by Sestrins and SAR1B. Furthermore, a recent study has also revealed that upon cellular entry, amino acids can be transferred via tRNA to inhibit K27-linked ubiquitination of mTOR mediated by GCN2-FBXO22 dephosphorylation [34–36]. BCAAs, especially leucine, participate in physiological processes such as mRNA transcription, metabolism, and protein deposition through amino acid sensing regulation mediated by mTOR [18, 37, 38]. Meanwhile, different ratios of the three amino acids have been found to exert antagonistic effects related to competition for catabolic and transporter enzymes, and further studies have attempted to find the optimal ratio among the three amino acids and achieve the maintenance of energy metabolism to promote growth and feed utilization in fish [2, 39, 40]. In this experimental analysis, we discerned that exogenous BCAAs regulate the recovery of starved common carp after nutrient limitation, that is, complex BCAAs can target *fosb* by regulating endogenous miR-203a in skeletal muscle, where *fosb* acts as a downstream regulator of AMPK to promote protein deposition and muscle development and thus repair muscle damage and prevent muscle loss. These results identified *fosb* as a key regulatory target for coordinating skeletal muscle energy expenditure and catabolism and muscle production, and the specific regulatory mechanisms of complex BCAAs were explored.

### 4.1. BCAAs Regulate Skeletal Myogenesis in a Dose-Dependent Manner

The process by which fish adjust to periods of nutrient scarcity and subsequent nourishment can be categorized into four distinct stages: stress, transition, acclimatization, and recovery [41]. We focused on the role of BCAAs in energy intake and protein deposition during the recovery phase in starved common carp and in the repair of damage to skeletal muscle. The present study showed that increases in the concentration of each dietary BCAAs significantly increased the body weight and muscle crude protein content of the fish in the experimental group and significantly promoted the thickening of dorsal muscle fibers. This finding suggested that BCAAs may alleviate energy limitation-induced muscle atrophy.

BCAAs supplementation stimulates amino acid sensors and promotes the translocation of mTOR to the cell periphery. During this process, mTORC1 is actively recruited to the lysosome via Rag GTPases (Rag guanosine triphosphatases), and Rheb (Ras homolog, MTORC1 binding) then further stimulates mTORC1 kinase activity [42]. Subsequently, key regulators downstream of mTORC1 involved in protein translation, autophagy, and cell growth fulfill their roles [43, 44]. Previous studies have shown that in different species of fish, appropriate BCAAs promote growth and development and improve the muscle quality to varying degrees via signaling pathways such as protein kinase B (AKT)/TOR-FoxO3a or TOR/S6K1 (ribosomal protein S6 kinase 1) coupled to MRF (myogenic regulatory factor) genes [45–47]. In this study, BCAAs significantly regulated the expression of genes involved in protein synthesis pathways, such as *ampk*, *mtor*, *eif4e*, *and ef*, and suppressed the expression of autophagy-related genes, such as *atg12*, *atg5*, *atg16*, *atg4b*, and *lc3b*, thus affecting the muscle protein deposition rate. In addition, different concentrations of BCAAs can promote the expression of genes related to myoblast proliferation and differentiation, further promote myoblast proliferation, differentiation, migration, and fusion, thereby increasing the number of myofibers in skeletal muscle.

AMPK, which is known as adenosine 5′-monophosphate (5′AMP [adenosine monophosphate])-activated protein kinase [48], is an AMP-activated serine/threonine kinase that senses energy changes in the body and increases its activity in response to energy limitation, that is, increases the AMP:ATP (adenosine triphosphate) or adenosine diphosphate (ADP):ATP ratio [49]. Upon activation, AMPK triggers the phosphorylation of EEF2K, an inhibitory regulator of protein elongation. The phosphorylated form of EEF2K subsequently inhibits the activity of the downstream effector, EF, which regulates protein translational elongation and inhibits the anabolic process of protein synthesis [50]. In addition, AMPK is able to regulate lipid and cholesterol synthesis and hexosamine synthesis [51]. Notably, there was a marked increase in the expression levels of genes regulating protein translation elongation in the AMPK-EEF2K pathway. These results illustrate that the addition of BCAAs to feed enhances the availability of nutrients, promotes nutrient metabolism, regulates skeletal muscle myogenesis and protein remodeling, and induces myogenesis.

### 4.2. BCAAs Regulate *fosb* Expression and Promote Skeletal Muscle Growth

Based on these previous histological results, the addition of BCAAs significantly increased the expression of *fosb* [23]. *Fosb* is a member of the FOS family and interacts with the JUN (Jun proto-oncogene, AP-1 transcription factor subunit) family to coencode leucine zipper proteins and thus regulate cell proliferation, differentiation, angiogenesis, and survival [52, 53]. As revealed in studies of mouse SCs, FOS proteins are significantly induced within hours after muscle injury and are involved in stem cell activation and muscle damage repair [21]. *fos* and *fosb* play active roles in myogenic differentiation, myotube fusion, and cell proliferation in bovine myosatellite cells [22, 54]. The miR-133a-mediated regulation of *fosb* inhibits bovine primary myoblast differentiation and apoptosis and effectively promotes cell proliferation [55]. In this study, BCAAs addition increased *fosb* expression in muscle, whereas skeletal muscle *fosb* disruption significantly inhibited AMPK-EEF2K signaling. These findings suggest that *fosb* may regulate the AMPK-EEF2K pathway rather than act as a downstream regulator of mTOR to affect muscle protein synthesis.

AMPK can phosphorylate FoxO family transcription factors and activate the transcription of their downstream molecules to regulate the expression of genes related to protein degradation pathways, including those that are involved in autophagy and the UPP [56]. Among these, *foxo3a*, a member of the FoxO transcription factor family, is involved in the positive transcriptional regulation of autophagy and muscle atrophy [57]. In this study, *fosb* knockdown significantly increased autophagic flux and promoted autophagy, and the same findings were observed after increased expression of *atrogin-1* and *murf-1*. Similarly, *atrogin-1* and *murf-1* promote myofibrillar protein degradation through the UPP. Previous studies have shown that *atrogin-1* is able to regulate *myod* expression in skeletal muscle under conditions of muscle atrophy [58]. Similarly, the expression of genes involved in muscle differentiation, such as *myod*, was found to be significantly downregulated in this study. However, further studies are needed to determine the exact mechanism by which BCAAs regulate the expression of *fosb*, a possible downstream regulator of AMPK that promotes skeletal muscle growth.

### 4.3. *fosb* Regulates Muscle Protein Synthesis Through the AMPK-EEF2K Pathway

Several studies have shown that translational elongation is one of the major rate-limiting and key regulatory steps in protein synthesis [59]. As a calcium/calmodulin-dependent enzyme, EEF2K exerts multiple regulatory effects on cellular energy and proliferation and is also stimulated by signaling downstream of AMPK [27, 60]. On the one hand, EEF2K can directly target and regulate EF, a rate-limiting enzyme in translational elongation, to negatively regulate the translational elongation of polypeptide chains to control the rate of protein synthesis [61]. The AMPK-EEF2K pathway was blocked by AICAR treatment alone, by the inhibition of *eef2k* alone, or by cotreatment with *fosb*, and supplementation with BCAAs reversed the negative regulatory effects of the inhibitor treatment on cell proliferation and shrinkage. These findings suggested that *fosb* can act as a downstream regulator of AMPK involved in the regulation of protein translation initiation and in the deregulation of energy-consuming processes by AMPK. On the other hand, EEF2K can form a complex with STAT3 (signal transducer and activator of transcription 3) and PKM2 (pyruvate kinase M1/2) to inhibit aerobic glycolysis and thus regulate cell proliferation [62]. The knockdown of *eef2k* can activate *eef2 and tgf-β1* (transforming growth factor beta 1), promote myofibroblast proliferation and differentiation, and reduce myofibroblast apoptosis [63]. This study suggested that *fosb* can be involved in promoting cell proliferation as an upstream regulator of *eef2k*. In summary, *fosb* regulates muscle protein synthesis through the AMPK-EEF2K pathway, controls skeletal muscle proliferation, and promotes skeletal muscle growth.

### 4.4. miR-203a Targets *fosb* to Regulate Translation Elongation

Noncoding RNAs, as important targetable single-stranded RNAs for posttranscriptional regulation, can be involved in various life activities [64, 65]. Among them, miRNAs have gradually become important regulators of the process of skeletal muscle growth. By exploring the abovementioned process, we found that miR-203a may be involved in the regulation of protein metabolism [23]. The findings indicate that the levels of miR-203a are negatively associated with both the levels of BCAAs and the expression of *fosb* in muscular tissues. In vivo miR-203a injection experiments revealed that the knockdown of miR-203a in skeletal muscle prolonged the translation of *fosb*-regulated proteins and inhibited autophagy, which resulted in substantial promotion of muscle differentiation and proliferation. Previous studies on miR-203 have focused on the targeted regulation of cell development. For example, transient expression of miR-203 enhanced the differentiation of pluripotent stem cells [21]. Furthermore, it inhibits myoblast proliferation and differentiation by targeting and inhibiting *c-jun* and *mef2c* as well as impeding the differentiation of fast muscle fibers by targeting dmrt2a (doublesex and mab-3 related transcription factor 2) [29, 66]. These results suggest that BCAAs can regulate miR-203a, which targets *fosb* to improve muscle mass in common carp.

## 5. Conclusion

**Figure 5:**
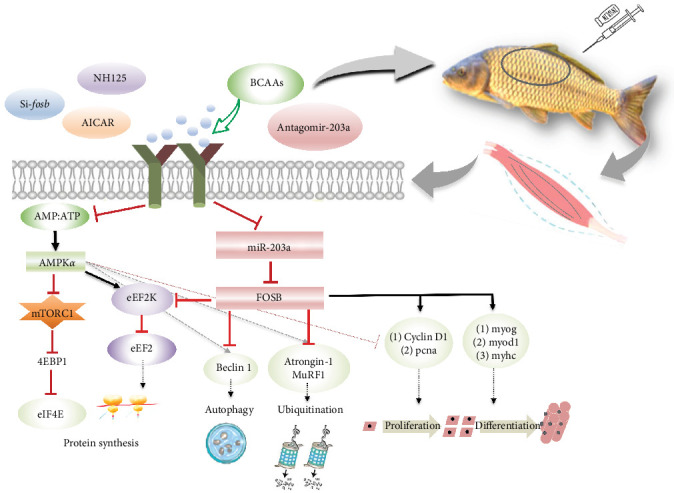
Involvement of BCAAs in the regulation of skeletal muscle growth and development in common carp. BCAAs, branched-chain amino acids.

Taken together, the addition of BCAAs to feed can support skeletal muscle protein remodeling and improve muscle mass by promoting protein deposition and myocyte proliferation and differentiation via miR-203a, which targets *fosb* (Figure 5). However, the regulatory mechanisms of non-coding RNAs in the modulation of skeletal muscle mass by BCAAs remain to be further explored in future research.

## Figures and Tables

**Table 1:** Composition of the diets used in this experiment (%).

Ingredients (%)	Dietary branched-chain amino acid levels (g/kg)			
	0	16	18	20
Fish meal^a^	15	15	15	15
Soybean meal	50	50	50	50
Rice bran	15	15	15	15
Wheat bran	8.3	8.3	8.3	8.3
Soybean oil	5.5	5.5	5.5	5.5
Vitamin mixture^b^	0.2	0.2	0.2	0.2
Mineral mixture^c^	2	2	2	2
Branched-chain amino acid premix	0	0.9	1.2	1.5
Gelatin	2	1.1	0.8	0.5
Ca(H_2_PO_4_)_2_	2	2	2	2
Total	100	100	100	100

^a^Fish meal: 90% dry matter, 53.5% crude protein, 10% crude lipid, and 20.8% ash.

^b,c^Vitamin and mineral mixture (IU or g/kg of mixture): Vit. A, 110,000 IU; Vit. D_3_, 320,000 IU; Vit. E, 2.4 g; Vit. B_1_, 1 g; Vit. B_2_, 2 g; Vit. B_3_, 7.8 g; Vit. B_6_, 1 g; Vit. B_12_, 125 mg; Vit. C, 18 g; folic acid, 0.4 g; D-biotin, 8 mg; potassium, 1.3 g, magnesium, 6 g; iron, 20 g; zinc, 3 g; copper,1.2 g; manganese,1 g; cobalt, 160 mg; iodine, 200 mg; selenium, 20 mg; inositol, 3 g; and moisture, 5% (Xi'an Lavia Biotechnology Co., Ltd.; Zhuhai Vino Breeding Co., Ltd.).

## Data Availability

The data that support the findings of this study are available from the corresponding author upon reasonable request.

## Nomenclature

miR-203a: microRNA-203a
siRNA: Small interfering RNA
AICAR: 5-Aminoimidazole-4-carboxamide1-*β*-D-ribofuranoside
BCAAs: Branched-chain amino acids
mRNA: Messenger RNA
miRNAs: MicroRNAs
fosb: FosB proto-oncogene, AP-1 transcription factor subunit
UPP: Ubiquitin proteasome pathway
eif4e: Eukaryotic translation initiation factor 4E
ef: Eukaryotic translation elongation factor 2 (eef2)
eif4ebp1: Eukaryotic translation initiation factor 4E binding protein 1
atg12: Autophagy related 12
atg5: Autophagy related 5
atg16: Autophagy related 16
atg4b: Autophagy related 4b
lc3b: Microtubule associated protein 1 light chain 3 beta (MAP1LC3B)
p62: Nucleoporin 62
psme2: Proteasome activator subunit 2
lamp2: Lysosomal associated membrane protein 2
beclin1: Beclin 1
phlpp1: PH domain and leucine rich repeat protein phosphatase 1
foxo3a: Forkhead box O3
myod1: Myogenic differentiation 1
myog: Myogenin
myhc: Myosin heavy chain 6
pcna: Proliferating cell nuclear antigen
AP-1: Activator protein-1
atrogin 1: F-box protein 32
murf1: Tripartite motif containing 101
Rag GTPases: Rag guanosine triphosphatases
Rheb: Ras homolog, MTORC1 binding
MRFs: Myogenic regulatory factors
S6K1: Ribosomal protein S6 kinase 1
JUN: Jun proto-oncogene, AP-1 transcription factor subunit
STAT3: Signal transducer and activator of transcription 3
PKM2: Pyruvate kinase M1/2
tgf-*β*1: Transforming growth factor beta 1
dmrt2a: Doublesex and mab-3 related transcription factor 2
mef2c: Myocyte enhancer factor 2C
AKT: Protein kinase B
AMP: Adenosine monophosphate
AMPK: Adenosine 5′-monophosphate (AMP)-activated protein kinase
ATP: Adenosine triphosphate
eEF2K: Eukaryotic elongation factor-2 kinase
mTORC1: Mammalian target of rapamycin complex 1
ADP: Adenosine diphosphate.

## Supporting Information

Supporting Information Table S1. RT-qPCR primers for mRNAs and miRNAs.
