# Supplementary material for: Branched-Chain Amino Acids Target miR-203a/fosb Axis to Promote Skeletal Muscle Growth in Common Carp (Cyprinus carpio)
Source: Aquac Nutr. 2025 Feb 26;2025:9406490. doi: 10.1155/anu/9406490 (PMC11882326; doi:10.1155/anu/9406490)
Supplement: Supporting Information — Table S1. RT-qPCR primers for mRNAs and miRNAs. [file 9406490.f1.docx]

**Supplementary materials**

Supplementary Table S1: RT-qPCR primers for mRNAs and miRNAs

| Primer | Acc. Num. | Oligonucleotide sequence (5′→3′) |
| --- | --- | --- |
| *myhc* | XM_019089518.2 | F: GGATGATGCTCTGCGTGGTAAT  R: TCAGCCAGTTTCCTTCCTCTCTC |
| *ampkα* | XM_042777568.1 | F: CAACATGAATGCCAAGATCGC  R: GTCATCAAATGGCAGAGTCCC |
| *ef* | XM_042724498.1 | F: GTTACAGATACAATGCGGTT  R: TCGCCCCATAATTTAGCC |
| *eef2k* | XM_042735113.1 | F: AAAGCGGAAAGAAGGGTGG  R: CTTGGTGCGGGTGTTGGTA |
| *mtor* | XM_042761448.1 | F: TGGGTGTTTCTTTTCCCTCGT  R: GTCCCGCACCCTGTTGATAA |
| *eif4ebp1* | XM_019077307.2 | F: TAACATTCCAGGGGTGACCA  R: CCCTGCGCTCTTGTCC |
| *eif4e* | XM_019114019.2 | F: TGGGCATTATACAACCAC  R: GACTCTCCGATTAAACACA |
| *atg12* | XM_042738226.1 | F: CGCCGAAATGATGGACTTCAC  R: TCTACTGTTGCCTCTGGACG |
| *fosb* | XM_042743892.1 | F: AGAAGAGCAACGACACCACAA  R: TTGTACGGCTGGTTCTTGGTT |
| *atg5* | XM_019093485.2 | F: CTATCAAGACCAGCCTCCC  R: CTTCACAGTCAAACCCACCACGGAG |
| *atg16* | XM_019092615.1 | F: TGGCTTGAAATTAGTGGACAG  R:ACAGGGAACCTTAAAGCGAGT |
| *lc3b* | XM_019072575.1 | F: GCCATCCGACAGACCCTT  R: TGAGCTGCAGTCTACGCC |
| *p62* | XM_042737795.1 | F: AAGACCAAGGCAGTGATGAGGAATG  R: GCTTGTGCTGGAGTCGGTACTTAG |
| *psme2* | XM_019089518.2 | F:GATCTCACAGCTGGACAACTT　R:AGAAACAGTGATGCAGGTCTC |
| *atg4b* | XM_042725815.1 | F: GTAATAGGTGGGAAGCCGAACAGTG  R: AGTGGTAGGAGTCGTCTGGGAAC |
| *lamp2* | XM_042770237.1 | F: TCTATGGGAGGCATCTGTCGGAAG  R: ACTTGTTGTTCTGGACTGCGAAGG |
| *phlpp1* | XM_042719321.1 | F: GCTGGAGGAGGAGGTGAAGGAG  R: AGAGCGGCGTGTCGTAGTAGTC |
| *beclin1* | XM_019078401.1 | F:TTCCTGCAGGTGACAAGTGAT  R:CCAGATGGTCCAGTAGTGCG |
| *foxo3a* | XM_019121026.2 | F: TGCTCTTCAGGCTACTCAGGATGG  R: ATTAGTGCGGGAACGGAAATCTGTC |
| *myod1* | XM_019068329.2 | F: AGAAATGCCATAAGTTACATCGAGT  R: ACCACTGAGCTTTTAGTATTCCGT |
| *myog* | XM_019096789.2 | F: CCAAACAGGATATCAGGACCGAA  R: ACAAGGACAGACCAAGGCTA |
| *ccnd1* | XM_042754412.1 | F: TTCCGCTGGCTATGAACTACCTG  R: TCCATTTCCAATAATTCGCTGGG |
| *pcna* | XM_019123359.2 | F: TCTGAAAGACCTCATCACCGAA  R: TATCTTCATTTCCAGCGCACT |
| *atrogin1* | XM_042741001.1 | F: GTCTGGTTCAGGACATGGGC  R: AGCTGTAAGCTAGCCGGG |
| *murf1* | XM_042751511.1 | F: AGGAGACCCTGGATCCTGAC  R: GTTGTCTCCGGTGTGGGATT |
| *rpl8* | XM_042731504.1 | F: TGTTGAGCATCCCTTCGG  R: GGTCTITGTTCCACGCAGT |
| ccr-mir-203a | MIMAT0026260 | F: GUGAAAUGUUUAGGACCACUUG  R: AACGCTTCACGAATTTGCGT |
| usb1 | XM_019068153.2 | F: ACGGCGCTTCGGCAGCACATATAC  R: AACGCTTCACGAATTTGCGT |

Sequences of the primers used in this experiment
